# Sedentary behavior modifies the effect of balance rehabilitation on balance discordance in Parkinson’s disease

**DOI:** 10.1038/s41531-026-01357-0

**Published:** 2026-04-16

**Authors:** Franziska Albrecht, Sarah J. Conklin, Andrew Hooyman, Daniel S. Peterson, Jason K. Longhurst, Erika Franzén

**Affiliations:** 1 Division of Physiotherapy, Department of Neurobiology, Care Sciences and Society, Karolinska Institutet, Stockholm, Sweden; 2 Women’s Health and Allied Health Professional Theme, Karolinska University Hospital, Stockholm, Sweden; 3 Department of Physical Therapy and Athletic Training, Saint Louis University, Saint Louis, USA; 4 Department of Physical Therapy, Chapman University, Irvine, USA; 5 College of Health Solutions, Arizona State University, Phoenix, USA

**Keywords:** Health care, Neurology, Neuroscience

## Abstract

Individuals with Parkinson’s disease (PD) often experience a misalignment between their perceived and actual balance ability, known as balance discordance, which has been associated with falls. We examined whether high-intensity balance and gait training (HiBalance) alters balance discordance in individuals with PD, and whether baseline sedentary behavior influences these changes. A secondary analysis examining pre- to post-intervention discordance changes of two HiBalance clinical trials (*N* = 97) using linear regression with interactions between sedentary behavior and pre-intervention discordance. The sample included two cohorts: one clinical and one research based. The model including sedentary behavior and its interaction with pre-intervention discordance explained 49% of variance. Significant predictors of post-intervention discordance were pre-intervention discordance (β = 8.78, *p* < 0.001) and cohort (β = 8.82, *p* = 0.006), while the interaction between pre-intervention discordance and sedentary time did not reach significance (β = −2.64, *p* = 0.05). Sensitivity analyses revealed that the clinical-based cohort model explained 24.7% of the variance in post-intervention discordance, with the interaction between pre-intervention discordance and sedentary time (β = −7.23, *p* = 0.004) as a significant predictor. HiBalance training did not significantly alter balance discordance. However, pre-intervention sedentary behavior may influence how much individuals with PD recalibrate the relationship between perceived and actual balance following physical rehabilitation.

## Introduction

Balance discordance is the alignment or malalignment between an individual’s actual and perceived balance ability^1,2^. Recent, retrospective studies have linked this outcome to fall risk, showing that individuals with Parkinson’s disease (PD) who are under-confident in their balance (negative discordance) are more likely to report falls compared to those who are over-confident (positive discordance)^1,2^. Beyond fall risk, balance discordance may have implications for physical activity engagement and participation in tasks with perceived balance demands. Comparable to balance discordance, mismatches between perceived and actual fall risk have been documented in older adults^3–6^. Historically, this work has classified individuals into quadrants based on whether individuals have high or low balance ability and high or low fear of falling or balance confidence, with each profile linked to distinct psychological characteristics and behavioral patterns^3,4^. For instance, older adults who perceived themselves to be at high risk of falling despite having low physiological fall risk experienced more falls and poorer function, likely due to maladaptive behaviors such as excessive fear or reduced activity^3^. Those who underestimated their fall risk remained active and reported better quality of life but were still vulnerable to falling^3^. Balance discordance represents a conceptually similar construct but captures the magnitude of this perceptual mismatch on a continuous scale rather than through predefined categories^1,2^. As with other forms of perceptual misalignment, “under-confidence” may contribute to unnecessary activity restriction, while “over-confidence” may lead to increased fall risk through engagement in risky activities. These perceptual mismatches may have important implications for safety, function, and participation.

Balance discordance in PD may also be associated with cognitive, perceptual, and mood-related factors, including perceived health, anxiety, and depressive signs. Specifically, individuals who report poorer perceived health and higher anxiety or depressive symptoms tend to underestimate their balance ability, exhibiting “under-confidence”^7^. Additionally, balance discordance may have a nonlinear relationship with cognitive performance^8^. Individuals with lower cognitive function are more likely to experience misalignment between perceived and actual balance ability, resulting in either overestimation or underestimation of their true capacity^8^. These psychological and cognitive associations may have meaningful implications for both physical function and quality of life in PD^9^. For example, elevated anxiety or depressive symptoms have been linked to greater fear of falling, avoidance behavior, and overprotective movement strategies^10,11^. These factors may further contribute to a cycle of inactivity, physical deconditioning, and further decline in motor function^12–14^.

Indeed, sedentary behavior is common among people with PD, despite clear evidence that physical activity improves both motor and non-motor symptoms and enhances quality of life^15–19^. Many individuals with PD fall short of recommended activity levels^20^, spending an average of 10 waking hours per day sedentary, often in longer uninterrupted bouts than age-matched individuals without PD^17^. While this inactivity is partly driven by mobility impairments^21^, psychological factors such as depression, cognitive impairment, and fear of falling also contribute substantially^12,22,23^. Fear of falling, for instance, leads to activity avoidance in up to 70% of individuals with PD^24,25^, underscoring the impact of psychological factors on physical activity behavior.

Despite the conceptual rationale suggesting perception and ability interact to shape daily activity choices, few studies have empirically assessed this relationship. To date, only one study has reported modest associations between balance discordance and objectively measured movement behaviors, including step count and sedentary time^7^. Importantly, physical activity and sedentary behavior represent related but distinct constructs. Consistent with this distinction, prior work has examined related psychological constructs in the context of physical activity outcomes. Previous research shows that individuals with moderate to high avoidance behavior due to fear of falling had lower average daily activity levels. Specifically, they accumulate fewer hours stepping per day, take fewer total daily steps, exhibit lower daily metabolic equivalents (METs) compared to individuals with low avoidance behavior due to fear of falling^26^. Moreover, it remains unclear whether changes in balance discordance can be achieved through intervention, and whether such changes are influenced by an individual’s baseline activity level or psychological profile.

Prior research has shown that balance and gait training can improve objective balance ability^27–29^ and balance confidence in people with PD^29,30^. These improvements are promising given that both actual ability and confidence play critical roles in daily activity, fall risk, and quality of life^31–36^. However, improvements in ability and confidence may not necessarily occur in parallel or at the same rate. An individual may demonstrate improved balance ability on clinical tests yet continue to report low confidence, or vice versa. Balance discordance captures this relationship and may provide a more nuanced indicator of whether balance interventions are targeting the perceptual-motor mismatch that contributes to activity restriction or fall risk in PD. Notably, prior work has shown that the HiBalance program, a highly challenging balance training program, can improve physiological balance performance without corresponding improvements in balance confidence^37^. This dissociation suggests that balance discordance may be sensitive to intervention-related change, as improvements in ability without parallel changes in perception could alter the alignment between the two. However, while changes in balance ability and balance confidence are often reported separately, the alignment between perceived and actual performance is rarely examined as an outcome, and it remains unclear whether such realignment occurs at the group level or only within specific subgroups. Thus, the primary aim of the present study was to determine whether a high-intensity balance and gait intervention changes balance discordance in individuals with PD. We hypothesized that the intervention would reduce balance discordance by improving the alignment between perceived and actual balance ability. Secondarily, we aimed to examine whether pre-intervention physical activity levels and sedentary behavior, treated as distinct constructs, were associated with changes in balance discordance following the intervention. We hypothesized that baseline physical activity and higher sedentary time would modify the pre to post intervention discordance relationship. Specifically, individuals with lower baseline physical activity and higher sedentary time would demonstrate greater improvements in balance discordance than those who already engage in higher levels of physical activity and lower sedentary time.

## Results

### Participants

The baseline data of a highly challenging balance and gait intervention (HiBalance) groups from two clinical trials (EXercise in PArkinson’s disease and Neuroplasticity (EXPANd) and the Effectiveness/Implementation cohorts) were merged. The two cohorts differed significantly in disease severity (Hoehn & Yahr scores, pre- and post-intervention), subjective balance (Activities-specific Balance Confidence (ABC), pre- and post-intervention), and close to significant in subjective health (EuroQol 5 Dimension survey visual analogue scale (EQ5-D VAS) post-intervention) (Table 1).

**Table 1:** Participant demographics and clinical characteristics for the Implementation and EXPANd cohorts, as well as the total sample

	*Implementation Cohort (N* *=* *57)*	*EXPANd Cohort (N* *=* *40)*	*Total (N* *=* *97)*	*p*
***Age*** *(years)*	71.0 (69.0, 72.9)	70.3 (68.6, 72.0)	70.7 (69.4, 72.0)	0.164^a^
***PD Duration*** *(years)*	6.8 (5.3, 8.3)	5.9 (4.5, 7.4)	6.4 (5.4, 7.4)	0.436^a^
***Sex***				0.101^b^
*Male*	26 (45.6%)	25 (62.5%)	51 (52.6%)	
*Female*	31 (54.4%)	15 (37.5%)	46 (47.4%)	
***Hoehn & Yahr (pre)***				< 0.001^b^
*Missing*	4	0	4	
*Stage 1*	6 (11.3%)	0 (0.0%)	6 (6.5%)	
*Stage 2*	20 (37.7%)	36 (90.0%)	56 (60.2%)	
*Stage 3*	27 (50.9%)	4 (10.0%)	31 (33.3%)	
***Hoehn & Yahr (post)***				< 0.001^b^
*Missing*	33	1	34	
*Stage 1*	4 (16.7%)	0 (0.0%)	4 (6.3%)	
*Stage 2*	10 (41.7%)	34 (87.2%)	44 (69.8%)	
*Stage 3*	10 (41.7%)	5 (12.8%)	15 (23.8%)	
***LEDD (pre****, mg****)***	580.0 (490.2, 669.9)	618.6 (500.6, 736.6)	597.6 (526.3, 668.8)	0.571^a^
*Missing*	9	0	9	
***LEDD (post****, mg****)***	-	641.8 (520.7, 762.9)	641.8 (520.7, 762.9)	NA
*Missing*	57	2	59	
***MiniBESTest (pre)***	20.6 (19.7, 21.5)	21.1 (20.0, 22.2)	20.85 (20.16, 21.54)	0.497^a^
*Missing*	4	0	4	
***MiniBESTest (post)***	22.6 (21.7, 23.5)	22.1 (21.1, 23.1)	22.39 (21.73, 23.05)	0.367^a^
*Missing*	4	1	5	
***TUG*** *(****pre****, seconds)*	10.6 (10.0, 11.2)	10.6 (9.8, 11.5)	10.61 (10.12, 11.10)	0.969^a^
*Missing*	4	0	4	
***TUG*** *(****post****, seconds)*	10.0 (9.5, 10.6)	9.9 (9.2, 10.6)	9.98 (9.58, 10.39)	0.987^a^
*Missing*	4	1	5	
***TUG-cog*** *(****pre****, seconds)*	18.1 (14.8, 21.5)	14.8 (13.0, 16.5)	16.59 (14.61, 18.56)	0.230^a^
*Missing*	10	0	10	
***TUG-cog*** *(****post****, seconds)*	13.7 (12.5, 14.8)	15.0 (12.4, 17.7)	14.3 (13, 16)	0.246^a^
*Missing*	8	1	9	
***Fall in the last 12 months (pre)***				0.383^b^
*Missing*	10	2	12	
*No*	24 (51.1%)	23 (60.5%)	47 (55.3%)	
*Yes*	23 (48.9%)	15 (39.5%)	38 (44.7%)	
***ABC (pre)***	66.5 (61.7, 71.3)	80.9 (75.8, 86.03	72.7 (68.9, 76.5)	<0.001^a^
*Missing*	4	0	4	
***ABC (post)***	66.4 (61.7, 71.1)	84.5 (80.5, 88.5)	74.5 (70.8, 78.2)	<0.001^a^
*Missing*	10	2	12	
***EQ-5D VAS (pre)***	62.54 (57.90, 67.18)	68.33 (63.58, 73.07)	65.05 (61.73, 68.38)	0.102^a^
*Missing*	5	0	5	
***EQ-5D VAS (post)***	67.98 (63.85, 72.11)	73.89 (69.14, 78.65)	70.58 (67.46, 73.70)	0.051^a^
*Missing*	10	3	13	
***Steps per day (pre)***	4580 (3922, 5238)	5570 (4747, 6393)	4998 (4483, 5513)	0.074^a^
*Missing*	5	2	7	
***Steps per day (post)***	4627 (3939, 5315)	5253 (4259, 6247)	4899 (4327, 5470)	0.419^a^
*Missing*	10	4	14	
***Sedentary time per day (pre****, minutes****)***	610.8 (582.7, 638.8)	617.1 (587.9, 646.2)	613.4 (593.5, 633.3)	0.769^a^
*Missing*	5	2	7	
***Sedentary time per day (post****, minutes****)***	596.7 (565.0, 628.4)	600.4 (569.6, 631.3)	598.3 (576.4, 620.3)	0.869^a^
*Missing*	10	4	14	
***LPA time per day (pre****, minutes****)***	170.3 (149.3, 191.2)	167.3 (143.0, 191.6)	169.0 (153.5, 184.6)	0.662^a^
*Missing*	5	2	7	
***LPA time per day (post****, minutes****)***	176.6 (154.4, 198.9)	171.9 (144.5, 199.4)	174.6 (157.6, 191.6)	0.673^a^
*Missing*	10	4	14	
***MVPA time per day (pre****, minutes****)***	35.6 (27.5, 43.8)	45.2 (36.2, 54.1)	39.7 (33.7, 45.6)	0.066^a^
*Missing*	5	2	7	
***MVPA time per day (post****, minutes****)***	34.1 (26.6, 41.6)	39.7 (29.3, 50.2)	36.5 (30.5, 42.6)	0.502^a^
*Missing*	10	4	14	
***Discordance (pre)***	5.00 (0.12, 9.88)	19.45 (14.42, 24.47)	11.21 (7.45, 14.97)	< 0.001^a^
*Missing*	4	0	4	
***Discordance (post)***	3.75 (−0.85, 8.35)	21.73 (17.66, 25.81)	11.67 (8.03, 15.31)	< 0.001^a^
*Missing*	10	3	13	

Values are presented as means (95% confidence intervals) for continuous variables or counts (percentages) for categorical variables.

^a^ Wilcoxon rank sum test.

^b^ Pearson’s Chi-squared test.

NA Not Applicable, PD Parkinson disease, LEDD Levodopa Equivalent Daily Dose, LPA Light Physical Activity, TUG Timed Up and Go, TUG-cog Timed Up and Go Cognitive, ABC Activities-Specific Balance Confidence Scale, MiniBESTest Mini-Balance Evaluation Systems Test, MVPA Moderate to Vigorous Physical Activity, pre Before intervention, post Immediately following intervention.

### Discordance and the HiBalance intervention

The paired t-test between pre- and post-intervention discordance of the whole HiBalance cohort was not statistically significant, indicating that the sample mean discordance levels did not change in response to participants completing the HiBalance intervention (t = −0.0042, df = 83, *p*-value = 0.997).

The linear model predicting post-intervention discordance accounted for 50.8% of the variance in discordance, with the intercept being significant (β = 8.47, CI = 3.91 – 13.03, *p* < 0.001) (Table S1). Pre-intervention discordance (β = 9.09, CI = 6.05 – 12.12, *p* < 0.001) and cohort (EXPANd) (β = 9.62, CI = 3.71–15.53, *p* = 0.002) were significant predictors. The other covariates of age, sex, and disease severity (Hoehn & Yahr) were not significant.

Next, exploratory interaction terms between pre-intervention discordance and 1) all accelerometer-derived movement behavior variables considered together (including steps per day, sedentary time, light physical activity (LPA), and moderate-to-vigorous physical activity (MVPA)); 2) steps per day; 3) sedentary behavior; 4) LPA; 5) MVPA; 6) subjective health (EQ5-D VAS); and, in a sub-cohort, 7) anxiety and depression (Hospital Anxiety and Depression scale (HADS)) were added to linear models. In models 2) and 4)-7), no significance was observed for variables of interest or their interaction terms.

The linear model 1) predicting post-discordance, using all accelerometer-based physical activity variables and sedentary behavior, showed multicollinearity. This was resolved with the removal of steps per day. The updated model, including sedentary time, LPA, and MVPA, accounted for 46.64% of the variance in discordance (Table S2). In this model, the intercept (β = 9.05, CI = 3.74 – 14.37, *p* = 0.001), pre-intervention discordance (β = 8.64, CI = 5.12 – 12.17, *p* < 0.001), and cohort (EXPANd) (β = 8.7, CI = 2.31 – 15.08, p = 0.008) were all significant predictors of post-intervention discordance.

**Fig. 1:**
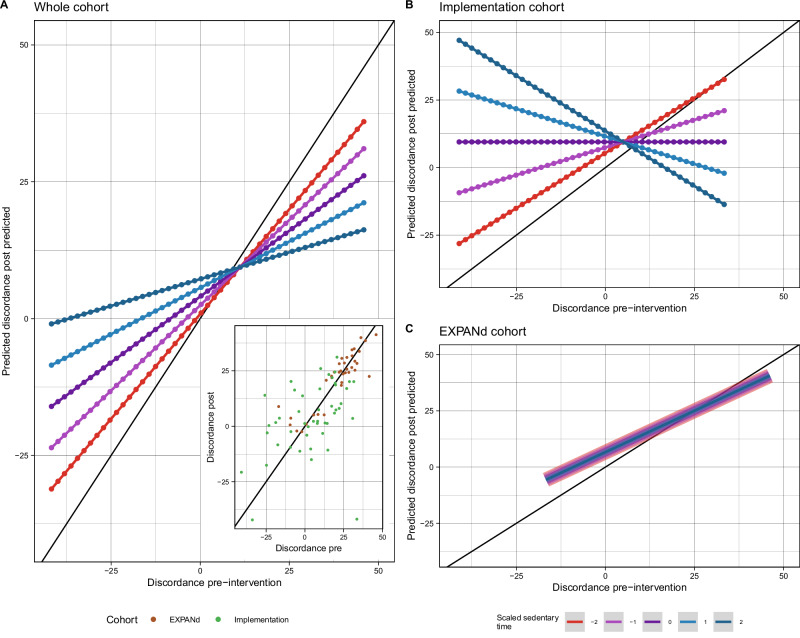
Modeled relationship between pre- and predicted post-intervention balance discordance predicted and their interaction with pre-intervention sedentary behavior. The black line represents perfect prediction, where pre-intervention discordance values would equal post-intervention discordance values (slope = 1, intercept = 0). Discrete levels of scaled sedentary time are based on simulated groups, assigned the same scaled discordance distribution as the data, spanning from −2.9 (most under‑confidence) to 1.9 (most over‑confidence) in 0.1 increments. **A** Combined cohort, with the small plot in the bottom right corner showing raw pre- to post-intervention discordance values colored by the cohort. In this model, higher pre-intervention discordance, higher sedentary time, and cohort membership predicted post-intervention discordance, with sedentary time moderating the pre–post relationship. **B** Implementation cohort, representing a more clinically-based population, visualized independently. Here, the interaction between pre-intervention discordance and sedentary time was a significant predictor. **C** EXPANd cohort, representing a more research-based population, visualized independently. In this model, only pre-intervention discordance significantly predicted post-intervention values, and the interaction with sedentary time was not significant.

Figure 1 illustrates the modeled relationship between pre- and post-intervention balance discordance and the moderating role of sedentary behavior. In the combined cohort, linear model 3 (with sedentary behavior as an interaction term) was significant and accounted for 49% variance in post-intervention discordance (Table 2, Fig. 1A). In this model, the intercept (β = 9.42, CI = 4.33 – 14.52, *p* < 0.001), pre-intervention discordance (β = 8.78, CI = 5.46 – 12.10, *p* < 0.001), and cohort (EXPANd; β = 8.82, CI = 2.64 – 15.00, *p* = 0.006) were significant predictors of post-intervention discordance. Further, the interaction between pre-intervention discordance and sedentary time was close to being significant (β = −2.64, CI = −5.27 – 0, *p* = 0.050). The interaction between pre-discordance and sedentary time shows that people who were least sedentary maintained post-discordance levels similar to their pre-discordance. As sedentary time increases, individuals with higher pre-discordance exhibit greater decreases in discordance, while those with low pre-discordance show the opposite trend, with post-discordance increasing alongside sedentary time.

**Table 2:** Linear regression predicting post-intervention discordance using an interaction of discordance pre-intervention with sedentary time

	Discordance post-intervention		
*Predictors*	*Estimates*	*CI*	*p*
(Intercept)	9.42	4.33 – 14.52	**<0.001**
Age	-2.49	-5.82 – 0.83	0.139
Sex	-3.19	-9.54 – 3.16	0.319
Cohort [EXPANd]	8.82	2.64 – 15.00	**0.006**
Hoehn & Yahr pre-intervention	1.04	-2.08 – 4.16	0.509
Discordance pre-intervention	8.78	5.46 – 12.10	**<0.001**
Sedentary time mean min per day pre-intervention	-0.22	-3.37 – 2.94	0.891
Discordance pre-intervention × Sedentary time mean min per day pre-intervention	-2.64	-5.27 – −0.00	**0.050**
Observations	77		
R2 / R2 adjusted	0.536 / 0.489		

Sensitivity analyses were conducted to determine whether effects were driven by one of the cohorts, where we split the two cohorts and rebuilt the models. For the Effectiveness/Implementation cohort (Fig. 1B), the model accounted for 24.7% of the variance in post-intervention discordance, and the interaction between pre-intervention discordance and sedentary time (β = −7.23, CI = −12.01 to −2.44, *p* = 0.004) emerging as a significant predictor (Table S3). In contrast, for the EXPANd cohort (Panel C), the model accounted for 75.6% of the variance in post-intervention discordance, with only pre-discordance being a significant predictor (β = 11.56, CI = 8.92–14.20, p < 0.001) (Table S4, Fig. 1). In this cohort, the interaction between pre-intervention discordance and sedentary time was not significant (*p* = 0.580).

## Discussion

In this sample, HiBalance, a highly challenging balance and gait training program, did not significantly impact discordance between perceived and actual balance ability at the group level. Therefore, despite previous reports of modest training effects on physical performance outcomes such as Mini-Balance Evaluation Systems Test (MiniBESTest) and gait velocity^27,37,38^, there were no observed changes in alignment of actual and perceived balance ability (measured via discordance) as a result of the intervention. However, in cohort-specific analyses, which examined the moderating effect of physical activity and sedentary time on the pre/post discordance relationship, sedentary time emerged as a significant moderator in the Effectiveness/Implementation cohort, such that individuals with greater pre-intervention sedentary time demonstrated larger reductions in discordance (i.e., became more concordant), whereas those who were less sedentary showed little change. This moderating effect was not observed in the EXPANd cohort.

The absence of a group-level change in balance discordance suggests that perceptual constructs underlying balance discordance may be more cognitively or psychologically anchored and less responsive to balance and gait-focused interventions alone. This may indicate that perceptual constructs like balance confidence are influenced not only by physical capability but also by factors such as previous experiences, fear of falling, and psychological characteristics like anxiety or depression. Given evidence that HiBalance improves balance performance without consistently improving balance confidence^37^, it is plausible that the intervention changes one component of discordance without sufficiently influencing the other, limiting net change at the group level. Other studies have suggested similar influences on perceptual outcomes, highlighting the complex and multifactorial nature of balance confidence and related constructs^12,39^. Further, these findings may indicate that interventions to improve alignment between perceived and actual balance may require elements beyond balance training alone. These elements would likely include the integration of psychological strategies based on cognitive behavioral therapeutic approaches which have shown to be effective at addressing fear of falling and other constructs similar to balance confidence^40–49^.

As stated before, while no overall group-level change in balance discordance was observed, there was a significant interaction between pre-intervention discordance and sedentary activity in predicting post-intervention discordance values in the Effectiveness/Implementation cohort. Individuals who were more sedentary prior to the intervention became more aligned in their perceived and actual balance ability (i.e., more concordant) following training, whereas those who were less sedentary showed minimal change. One possible explanation is that sedentary individuals were exposed to a level of balance challenge and performance feedback that differed substantially from their recent daily experience, creating greater opportunity for recalibration of perceived ability. This interpretation aligns with previous responder analyses of the HiBalance program showing larger training-related gains among individuals with poorer baseline physical and cognitive performance^50^.

The observed moderating effect of sedentary behavior is consistent with principles from Bandura’s self-efficacy theory, which emphasizes the role of mastery experiences in shaping perceived ability^51^. Individuals who were more sedentary prior to the intervention may have had fewer recent opportunities to successfully engage in challenging balance tasks, making the HiBalance program a relatively novel and salient source of performance feedback. Successfully completing demanding balance and gait tasks may therefore have facilitated recalibration of perceived balance ability in this subgroup, contributing to greater alignment between perceived and actual balance. In contrast, individuals who were less sedentary may have already developed more stable perceptions of their balance abilities through regular activity and exercise. Because the intervention may not have provided challenges or feedback substantially different from those encountered in daily life, and because these individuals may have developed effective coping or compensatory strategies, there may have been limited impetus for perceptual change during the intervention.

As stated, post hoc analyses revealed cohort differences, with the Effectiveness/Implementation cohort demonstrating a stronger interaction between pre-intervention discordance and sedentary time than the EXPANd cohort, despite both cohorts receiving the same intervention. These findings may reflect inherent differences in the characteristics of the two cohorts. The Implementation cohort included a more clinically complex population, with higher disease severity and lower baseline balance confidence, as indicated by differences in Hoehn & Yahr stage and ABC scores at baseline. In contrast, the EXPANd cohort was generally healthier, in part due to study eligibility criteria requiring MRI suitability, which excluded individuals with more advanced disease or comorbidities. These differences suggest that baseline clinical profile may influence the degree to which perceptual–motor alignment is amenable to change and highlight the importance of tailoring interventions to more complex clinical populations.

The findings of this study have clinical implications for the design and implementation of balance interventions in individuals with PD. Balance and gait training alone may be insufficient to address malalignment between balance ability and confidence, particularly in individuals who are already less sedentary. Incorporating psychological strategies, particularly those rooted in cognitive-behavioral therapy, may enhance intervention effectiveness by directly targeting balance confidence and related perceptual constructs such as fear of falling^40–49^. Conversely, individuals that are generally less active may respond more strongly to progressive balance training that introduces novel challenges and feedback, potentially creating opportunities for recalibrating self-perception of balance. Combining physical and psychological approaches may be beneficial across activity levels to promote alignment between perceived and actual balance ability and reduce activity avoidance.

Several limitations should be considered when interpreting the results of this study, including issues related to generalizability and study design. The study was conducted in a Swedish sample within a region-specific rehabilitation setting, which may not reflect services, cultural contexts, or healthcare systems in other countries, thereby limiting external validity. Additionally, due to the study design and the highly challenging nature of the interventions, only ambulatory individuals with PD were included, and the findings may not generalize to individuals with more advanced disease stages or substantial mobility limitations. From a methodological perspective, the sample size may have limited statistical power to detect small group-level changes in balance discordance, as well as the observed statistically significant interaction effects. Future studies may benefit from examining individual-level trajectories of discordance change to better capture heterogeneity in treatment response. Although heterogeneity between cohorts may further limit generalizability, post hoc analyses revealed meaningful differences in response patterns between cohorts that, underscoring the potential relevance of baseline clinical characteristics. Larger and more diverse samples will be needed to more clearly delineate how clinical profile influences responsiveness of balance discordance to intervention. Next, because control groups were not included in the present analysis, observed changes may partially reflect regression to the mean rather than true intervention effects. Potential sources of bias should also be acknowledge, including selection bias related to recruitment procedures and measurement bias associated with the assessment methods and their reliability. Finally, multiple statistical tests were conducted across seven interaction models, increasing the risk of type I error; therefore, these findings should be interpreted with appropriate caution.

This study found that highly challenging balance and gait training did not produce significant changes in balance discordance in individuals with PD. However, among individuals with higher baseline sedentary behavior in the Effectiveness/Implementation cohort, balance discordance shifted toward greater alignment, suggesting greater responsiveness to novel balance challenges. This moderating effect was not observed in the EXPANd cohort, indicating that clinical complexity and disease severity may influence intervention outcomes. These findings highlight the importance of tailoring balance rehabilitation to individual sedentary behavior profiles and integrating psychological strategies alongside physical training to more effectively address misalignment between perceived and actual ability.

## Methods

### Participants

The present study is a secondary analysis of two merged datasets, one primarily clinical (Effectiveness/Implementation) and one primarily research based (EXPANd). using baseline data of the highly challenging balance and gait intervention (HiBalance) groups of these two trials, which were overseen by the same investigator team included a total of 97 participants with PD. In case of missing values, participants were not included in the analyses. All participants provided written informed consent. The studies were conducted in accordance with the Declaration of Helsinki. The Ethical Review Authority in Sweden approved the data merging and research questions (2023-02470-01). Detailed inclusion and exclusion criteria are provided below.

### Data

The *EXPANd* (EXercise in PArkinson’s disease and Neuroplasticity) *cohort* (*n* = 40) is derived from a randomized controlled trial conducted between 2018 and 2019. The trial was registered at ClinicalTrials.gov (NCT03213873, 2017-07-10) and approved by the Regional Ethical Review Board in Stockholm (2016/1264–31/4, 2017/1258–32, 2017/2445–32). Inclusion criteria comprised idiopathic PD diagnosis, age ≥60 years, Hoehn & Yahr stage 2 or 3, stable anti-Parkinson medication regimen, ability to walk unaided, Montreal Cognitive Assessment (MoCA) score ≥21, and Mini Balance Evaluation Systems Test (Mini-BESTest) score ≤27. Exclusion criteria included other balance, speech, or voice disorders; recent speech/balance programs; blindness; claustrophobia; severe tremor; dyskinesia; diplopia; dystonia; or Magnetic Resonance Imaging-incompatible implants. All participants were residents of Sweden^52^.

The *Effectiveness/Implementation cohort (n* = *57)* originated from a clinical trial with data collected between 2016 and 2017. The trial was registered at ClinicalTrials.gov (NCT02727478, 2016-03-29) and approved by the Regional Ethical Review Board in Stockholm (2016/201-31/12). Inclusion criteria were a diagnosis of idiopathic PD, cognitive ability to follow verbal instructions, ability to walk unaided, and no participation in structured exercise programs in the past 6 months. All participants were residents of Sweden^37^. The sample size of the present study differs from preceding publications. In the present study, we included participants with Hoehn & Yahr 1 and did not impute data^37,53^.

The same highly challenging balance and gait rehabilitation intervention *(HiBalance)* was implemented in both studies. This intervention consisted of 1-hour group sessions, held twice weekly for 10 weeks. Additionally, participants engaged in a prescribed home exercise program for 1 h each week. The intervention and home exercise program targeted four key components of balance control affected in PD: stability limits, anticipatory postural adjustments, sensory integration, and motor agility, following motor learning principles such as specificity, progressive overload, and variation. The program also included dual-task exercises that progressively integrated cognitive tasks (e.g., counting or memory tasks) with motor tasks (e.g., carrying or manipulating objects). To ensure the exercises remained highly challenging, each task was individually adjusted by modifying the base of support, increasing movement speed/amplitude, restricting vision, and varying the degree of multitasking. The difficulty level was escalated in three consecutive blocks within each session and between sessions over the 10-week intervention period (see^37,52,54,55^ for intervention details). The intervention was performed in small groups (6-8 participants) and led by two trained physical therapists. Adherence to the home exercise program was logged by the participants and considered high with 82% in the HiBalance intervention group^55^.

Demographic data extracted included age, sex, PD severity (Hoehn & Yahr), and reported falls from the 12 months before the intervention. Participants were classified as fallers if they reported one or more falls in the previous 12 months. A description of sample characteristics is presented in Table 1.

Balance ability and functional mobility were assessed using the Timed Up and Go (TUG) test, which evaluates the performance of a sequential motor task involving rising from a chair, walking 3 m, turning, and returning to the chair. The TUG demonstrates high reliability in PD (ICC [3,1] = .87-.99) and is sufficiently sensitive to detect changes in performance^56^.

Balance ability was further assessed using the MiniBESTest, a valid and reliable measure for people PD, with an ICC of 0.80^57,58^. The 14-item MiniBESTest evaluates four domains: anticipatory balance, reactive postural control, sensory orientation, and dynamic gait. It has a maximum score of 28, with higher scores indicating better balance.

The ABC scale was used to measure perceived balance. It is a self-report tool assessing fall-related self-efficacy across 16 activities^59^. The score is expressed as a percentage of balance confidence. The ABC has shown satisfactory psychometric properties in PD, effectively capturing balance confidence^60^.

Physical activity levels and sedentary behavior were monitored by an Actigraph GT3X+ accelerometer (Pensacola, FL) placed on the hip over a span of 7 consecutive days. Participants were instructed to wear it for seven consecutive days, from getting up in the morning until going to bed, removing it only at night and during showering or bathing. The belt was adjusted to the waist and worn either on the skin or over clothing, with the sensor positioned on the side over the hip; it operated automatically without being switched on or off. Each day, the following information was to be recorded in a diary: the date, the times when the belt was put on and taken off, and any additional periods during the day when it was removed (e.g., for bathing).

The data, processed with ActiLife software, included sedentary time, step count, time in LPA, and MVPA. Activity intensity was classified using established count-per-minute (cpm) thresholds: sedentary time ( < 100 cpm), LPA (100–1040 cpm), and MVPA ( ≥ 1041 cpm). The device measures acceleration in three dimensions, with validated thresholds for total energy expenditure^61^. Data from at least 4 days, up to a maximum of 7 days, were considered, excluding any days with less than 540 min of wear time according to literature guidelines^62^.

The EQ5-D-VAS assessed perceived health. Respondents are asked to report their perceived health today with a value between 0 (the worst possible health status) to100 (the best possible health status)^63^.

The HADS measured anxiety and depression. It is a self-report measure that is an acceptable and valid scale for use in people with PD^64^. The 14-item scale consists of seven items forming the anxiety and seven items forming the depression subscale^65^.

Balance discordance was quantified as the discrepancy between observed and predicted ABC values for each subject, utilizing a multivariable regression model that correlates individual TUG times with ABC values, adjusting for age, sex, and disease duration. This modeling approach has been described in two prior publications in individuals with PD^1,2^. The original model was derived in one PD cohort^2^ and subsequently replicated in a second cohort^1^, which corresponds to the dataset used in the present study. A positive discordance value indicates that perceived balance exceeds predicted balance (“over-confident”), whereas a negative discordance value indicates that perceived balance is less than predicted balance (“under-confident”).

1 $${Predicted\; ABC}=80.7+(-1.8* {TUG})$$

2 $${Balance\; discordance}={Actual\; ABC}-{Predicted\; ABC}$$

### Statistical Analysis

We compared demographic, clinical, and motor variables between the cohorts using Wilcoxon Rank Sum test or chi-squared tests^66^.

To analyze the changes in discordance between the pre- and post-intervention periods, a paired t-test was used to compare the mean differences. Additionally, linear regression analysis was conducted to predict post-intervention discordance based on scaled pre-intervention discordance with the covariates scaled participants’ pre-intervention age, sex, cohort, Hoehn & Yahr. This provides insights into the predictive power of initial discordance levels on subsequent changes.

To assess which variables influence the prediction of post-intervention discordance, separate linear regression models for each variable of interest were utilized. The models included the covariates scaled participants’ pre-intervention age, sex, cohort, Hoehn & Yahr, the main effect of the variable of interest, and the interaction between scaled pre-intervention discordance and scaled variables of interest. These included movement behavior variables (steps per day, sedentary behavior, LPA, MVPA), subjective health (EQ5-D-VAS), and in a subcohort (EXPANd), anxiety and depression (HADS). All variables were scaled to prevent structural collinearity in the regression analyses, i.e. collinearity between the interaction term and main effects.

We additionally built a model that included all movement behavior variables (steps per day, sedentary behavior, LPA, MVPA) to investigate how these influence each other and their relationship to post-intervention discordance. The model included also covariates scaled participants’ pre-intervention age, sex, cohort, Hoehn & Yahr. Multicollinearity was checked using Variation Inflation Factor (VIF). A VIF higher than 10 was seen as an indicator of multicollinearity and such variables were further inspected for exclusion.

In case of a significant effect of the variable of interest, we ran sensitivity analyses and split the cohorts to build linear regression models for the cohorts separately. The rationale behind this was to address slight differences in cohort characteristics, including disease severity and study purposes—one more clinically oriented and one more research-oriented. This ensures that the observed effect is not driven by one cohort behaving differently in unexpected ways, improving confidence that the findings are robust and generalizable.

Significant linear models were plotted and visually inspected for homoscedasticity, normality of residuals, influential observations/outliers using Cook’s distance of 0.5 as the outlier threshold and multicollinearity.

To visualize the interaction between pre-intervention discordance and sedentary behavior, we generated a simulated prediction grid using the range of continuous discordance values grouped into distinct sedentary behavior thresholds (Fig. 1). Specifically, we created a data frame with 5 simulated sedentary behavior groups, with each group representing discrete levels of scaled sedentary behavior: −2, -1, 0, 1, 2, where 0 represents mean sedentary activity, −2 represents a least sedentary group and 2 represents the most sedentary group. Individual data points among these simulated groups are then assigned the same scaled discordance range with values ranging from -2.9 (most underconfident) to 1.9 (most overconfident), with the increments increasing by 0.1. These simulated data points are then passed through the fitted model on the study sample data, which used sedentary activity as a continuous variable, to generate predicted discordance values. The study sample model used on the simulated data for visualization:

3 $${Post\; intervention\; discordance}=13.5+\left(10.4-2.85* {sedentary\; time}\right)* {Pre\; intervention\; discordance}$$

This allowed us to visualize how distinct values of sedentary behavior modified, i.e. changed the underlying relationship, between a specific pre-intervention discordance to post-intervention discordance. To facilitate interpretation, the standardized discordance values were rescaled to their original metric using the transformation:

4 $${Unstandardized\; discordance}=\left({Standardized\; discordance}* 18.25903\right)+11.2141$$

The predicted values were visualized using a scatter plot with a linear smoothing line for each level of sedentary behavior. The x-axis represented the unscaled pre-intervention discordance, and the y-axis showed the model predicted discordance. A reference line with a slope of 1 and an intercept of 0 was added to indicate perfect prediction.

All analyses were run in RStudio (R version 4.4.0 (2024-04-24)). R script is available on OSF (osf.io/hrx6d/).

## Supplementary information

Supplementary Information

## Data Availability

With respect to the Swedish and EU personal data legislation (GDPR), data are not publicly accessible due to regulations regarding personal integrity in research, public access and privacy. The data are available on a reasonable request. Any sharing of data will be regulated via a data transfer and user agreement with the recipient.

## Code availability

The underlying code for this study is available in OSF and can be accessed via this link [https://osf.io/hrx6d].
