# Supplementary material for: Sedentary behavior modifies the effect of balance rehabilitation on balance discordance in Parkinson’s disease
Source: NPJ Parkinsons Dis. 2026 Apr 16;12:98. doi: 10.1038/s41531-026-01357-0 (PMC13087008; doi:10.1038/s41531-026-01357-0)
Supplement: Supplementary file 1 — Supplementary Information [file 41531_2026_1357_MOESM1_ESM.pdf]

Supplementary Information

Table S1 Multiple linear regression using only pre-intervention discordance and covariates.

	Discordance post-intervention		
Predictors	*Estimates*	*CI*	*p*
(Intercept)	8.47	3.91 – 13.03	**<0.001**
Age	-1.92	-4.79 – 0.96	0.189
Hoehn & Yahr pre-intervention	0.79	-2.04 – 3.62	0.579
Cohort [EXPANd]	9.62	3.71 – 15.53	**0.002**
Sex [female]	-2.26	-7.71 – 3.19	0.411
Discordance pre -intervention	9.09	6.05 – 12.12	**<0.001**
Observations	84		
R^2 / R^2 adjusted	0.538 / 0.508		

Table S2 Multiple linear regression including all physical activity variables except for steps per day due to multicollinearity.

	Discordance post-intervention		
Predictors	*Estimates*	*CI*	*p*
(Intercept)	9.05	3.74 – 14.37	**0.001**
Age	-2.46	-6.17 – 1.25	0.190
Hoehn & Yahr pre-intervention	1.19	-2.04 – 4.43	0.464
Cohort [EXPANd]	8.70	2.31 – 15.08	**0.008**
Sex [female]	-2.87	-9.56 – 3.82	0.394
Discordance pre-intervention	8.64	5.12 – 12.17	**<0.001**
Sedentary time mean min per day pre-intervention	-0.66	-4.37 – 3.05	0.723
Moderate to vigorous physical activity mean min per day pre-intervention	0.45	-3.77 – 4.67	0.832
Light activity time mean min per day pre-intervention	-1.54	-6.11 – 3.03	0.503
Discordance pre × Sedentary time mean min per day pre-intervention	-2.50	-5.97 – 0.97	0.155
Discordance pre × Moderate to vigorous physical activity mean min per day pre-intervention	1.56	-2.65 – 5.77	0.462
Discordance pre × Light activity time mean min per day pre-intervention	-0.63	-6.30 – 5.05	0.826
Observations	77		
R^2 / R^2 adjusted	0.544 / 0.466		

Table S3 post-hoc linear regression only in IMPLEMENTATION group.

	**Discordance post-intervention**		
Predictors	*Estimates*	*CI*	*p*
(Intercept)	9.55	1.72 – 17.37	**0.018**
Age	-2.91	-8.95 – 3.13	0.334
Sex [female]	-7.10	-18.16 – 3.96	0.201
Hoehn & Yahr pre -intervention	0.31	-4.61 – 5.23	0.898
Discordance pre -intervention	4.23	-1.23 – 9.70	0.125
Sedentary time mean min per day pre-intervention	-1.38	-6.93 – 4.16	0.616
Discordance pre × Sedentary time mean min per day pre-intervention	-7.23	-12.01 – -2.44	**0.004**
Observations	42		
R2 / R2 adjusted	0.357 / 0.247		

Table S4 post-hoc linear regression only in EXPANd group.

	Discordance post-intervention		
Predictors	*Estimates*	*CI*	*p*
(Intercept)	20.95	18.11 – 23.80	**<0.001**
Age	-1.08	-3.30 – 1.14	0.328
Sex [female]	-0.87	-5.59 – 3.85	0.710
Hoehn & Yahr pre-intervention	1.90	-0.56 – 4.37	0.125
Discordance pre-intervention	11.56	8.92 – 14.20	**<0.001**
Sedentary time mean min per day pre-intervention	0.69	-1.61 – 3.00	0.544
Discordance pre × Sedentary time mean min per day pre-intervention	-0.69	-3.23 – 1.84	0.580
Observations	35		
R2 / R2 adjusted	0.799 / 0.756		
